# Nasogastric tube, a warning sign for high-flow nasal cannula failure in infants with bronchiolitis

**DOI:** 10.1038/s41598-020-72687-z

**Published:** 2020-09-28

**Authors:** Milena Siciliano Nascimento, Danielle E. R. Quinto, Gisele C. Z. Oliveira, Celso M. Rebello, Cristiane do Prado

**Affiliations:** Pediatric Intensive Care Unit, Department of Pediatrics, Hospital Israelita Albert Einstein, Albert Einstein Avenue, 627-701, São Paulo, SP 05651-901 Brazil

**Keywords:** Respiratory tract diseases, Risk factors

## Abstract

High-flow nasal cannula (HFNC) therapy is routinely used in the treatment of infants with bronchiolitis. This study sought to identify markers associated with failure of HFNC therapy that serve as warnings for early staging of other ventilatory support products. A retrospective study of infants with a diagnosis of bronchiolitis, receiving HFNC and admitted to the pediatric intensive care unit from January 2016 to June 2017, was conducted. The subjects were divided into two study groups according to the success or failure of HFNC therapy. Risk factors were assessed using the following variables: age, time between hospital admission and start of HFNC, equipment model, and the need for a nasogastric tube. Eighty-one infants were studied, and 18 (21.7%) of them exhibited therapy failure. The results of the logistic models showed that the chances of failure for patients requiring a nasogastric tube during HFNC use were more likely than those for patients with oral nutrition (OR = 8.17; 95% CI 2.30–28.99; *p* = 0.001). The HFNC failure was not associated with the device used (OR = 1.56; 95% CI 0.54–4.52; *p* = 0.41), time between hospital admission and HFNC installation (OR = 1.01; 95% CI 0.98–1.03; *p* = 0.73), or age (OR = 0.98; 95% CI 0.82–1.17; *p* = 0.82). Among late outcomes evaluated, the patients with therapy failure had longer total durations of O_2_ use (*p* < 0.001) and longer hospital stays (*p* < 0.001). The need to use a nasogastric tube during HFNC use was associated with HFNC therapy failure and can be considered as a marker of severity in children with bronchiolitis.

## Introduction

Treatment of bronchiolitis is based on symptoms and includes oxygen therapy, nutrition and hydration. The type of ventilatory support selected is based on increasing degrees of respiratory failure, namely, conventional oxygen therapy, high-flow nasal cannula (HFNC) use, noninvasive ventilation (NIV) with continuous positive airway pressure (CPAP) and, finally, invasive mechanical ventilation (IMV)^1–3^.

HFNC therapy has emerged as a novel method to deliver warm, humidified air flow to provide noninvasive respiratory support with a titratable oxygen fraction that can generate a positive distending pressure with increased functional residual capacity and reduced breathing effort^4–6^. This procedure is easier to use, better tolerated, and more comfortable for the patient when compared with nasal CPAP^7,8^.

The use of HFNC therapy has been associated with improved washout of the nasopharyngeal dead space, better mucociliary clearance of the lung, and a more reliable oxygen supply compared with other oxygen delivery systems^9^. HFNC has also been shown to reduce intubation rates and to improve oxygen saturation levels and is associated with decreases in E_tCO2_ (end-tidal carbon dioxide) and the respiratory rate (RR) in children with bronchiolitis in Intensive Care Units (ICUs)^10,11^.

In the literature, some authors have studied physiological variables such as heart rate (HR), RR, inspired fraction of oxygen (FiO_2_), and oxygen saturation (SpO_2_) to determine HFNC therapy failure^12–15^. Although the behaviors of variables such as HR, RR, and FiO_2_ after initiation of HFNC therapy are related to the success or failure of therapy, no consensus is available regarding the maximum acceptable values^12–16^, and this lack of agreement makes the use of these variables quite subjective for application in clinical practice.

This study aims at identifying nonphysiological markers (different from RR, FiO_2_, SpO_2_) associated with the failure of HFNC therapy that could be used during an early stage to identify therapeutic failure and early escalation for ventilatory support. To date, few studies have discussed the nonphysiological risk factors associated with failure of HFNC therapy. Variables such as the need to use a nasogastric tube during HFNC use^17^, age^18^, and equipment model^19^ were already studied in attempts to determine an association with severity of respiratory failure: however, none of them has studied the association with HFNC therapy failure. Our hypothesis is that the need to use a nasogastric tube during HFNC use is a marker of HFNC therapy failure.

## Methods

### Study type and site

A retrospective observational study was carried out through analysis of the medical records of children who met the inclusion criteria after approval by Albert Einstein Hospital’s research ethics committee and is registered under CAAE number 77279317.4.0000.0071; the informed consent term was waived by the ethics and research committee of the Hospital Israelita Albert Einstein. All research was performed in accordance with relevant guidelines/regulations. This study received no funding.

### Inclusion criteria and dependent variables

The sample included children younger than 2 years of age with primary diagnosis of bronchiolitis and who received an HFNC as the first-choice treatment for respiratory failure. All patients were admitted to the pediatric intensive care unit of a private hospital in the city of São Paulo from January 2016 to June 2017. A patient’s medical record number was used, each patient had an extensive revision of the medical record and data were collected by a physical therapist.

The analyzed variables included age, sex, weight on admission, PIM2, M-WCAS score, need for sedation, the need to use a nasogastric tube during HFNC use, equipment model, duration of HFNC use, time between hospital admission and start of HFNC, total oxygen time, and length of hospital stay. The M-WCAS is a score comprising five topics: SpO_2_, inspiratory breath sounds, expiratory wheezing, accessory muscles and cerebral functions^20^. The score was measured immediately before the HFNC installation. The protocol of nasogastric tube use indication is based on clinical evaluation by the physician according to the severity of respiratory failure. This evaluation follows subjective criteria of the physician and does not include a specific value of RR or SpO_2_. The first attempt is always for the oral feeding. Infants are often evaluated with respect to oral ingestion, and those who did not maintain adequate ingestion have a medical indication for the use of a nasogastric tube.

### Primary and secondary outcomes

The primary outcome was considered to be failure of HFNC therapy, and the secondary outcomes were time of oxygen use and length of hospital stay.

### Criteria for HFNC therapy failure

As in our institution, criteria for HFNC therapy failure were not yet established (one of the reasons for the study), and failure of HFNC therapy was based on evaluation by the assisting team, being defined as the need for NIV (defined as using BIPAP or CPAP) or IMV.

### High-flow equipment

One of the two systems available in the pediatric ICU was used to perform HFNC therapy: the Optiflow system (Fisher & Paykel Healthcare, Auckland, New Zealand) or the Precision Flow system (Vapotherm, New Hampshire, USA). The Optiflow system was used in association with the babypap 1150-S (Fanem Ltda, Guarulhos—Brazil). Four sizes of Optiflow junior nasal cannulas (Fisher and Paykel Healthcare) were used as appropriate for each patient: OPT312 premature, OPT314 neonatal, OPT316 infant, and OPT 318 pediatric. The Precision Flow system was used with cannulas of 5 different sizes: neonate, infant, small pediatric, and pediatric. The protocol was based on a flow rate of 2.0 L kg^−1^ min^−1^ for patients up to 10 kg, and for patients greater than 10 kg, flow rates of 2.0 L kg^−1^ min^−1^ to the tenth kilogram and 0.5 L kg^−1^ min^−1^ after the eleventh kilogram were used.

### Statistical analysis

Categorical variables are expressed as absolute frequencies and percentages. Numerical variables were assessed for their distributions using boxplots and are expressed as medians and quartiles due to the presence of asymmetric distributions and outliers. The results are presented for the overall data and according to the failure or success of HFNC therapy.

To compare the profiles of patients with and without HFNC failure, chi-square, Fisher's exact and Mann–Whitney hypothesis tests were used as appropriate.

The Mann–Whitney tests were used to compare the profiles of patients with and without the need for use of a nasogastric tube during the HFNC. We also investigated the associations between age, time between hospital admission and start of HFNC, equipment model and the need for a nasogastric tube during HFNC therapy with failure to use the HFNC through logistic regression models. The results were presented for odds ratios, 95% confidence intervals for odds ratios (95% CI) and *p-*values. Associations of failure occurrence with factors were investigated through logistic models. The analyses were performed with SPSS software (SPSS version 13.0, SPSS, Chicago, Illinois) considering a significance level of 5%.

## Results

Of the 1749 children admitted during the study period, 363 (20.8%) received a primary diagnosis of bronchiolitis. For these 363 patients, the first choice respiratory support was room air for 75 (20.7%), conventional oxygen therapy for 192 (52.9%), the need to use NIV for 11 (3.0%), the need to use IMV for 2 (0.5%), and HFNC therapy for 83 (22.9%). HFNC therapy failure occurred in 18 patients (21.7%), 17 (20.5%) required NIV, 5 (6.0%) required IMV, and 4 required both IMV and NIV.

Table 1 presents the main demographic characteristics and main clinical interventions of the patients overall, according to HFNC success or failure. No differences in demographic characteristics were observed between the groups; however, the patients with failure used more midazolam (*p* < 0.001) and fentanyl (*p* < 0.002). In the analysis of the total duration of HFNC therapy, the patients with failure used the HFNC for less time compared with the success group: 12.8 h versus 56.8 h (*p* < 0.001).

**Table 1:** Demographic characteristics and main clinical interventions of the subjects overall and according to the success or failure of HFNC therapy. Values are presented as the median and interquartile range.

Variables	Total (n = 83)	Success (n = 65)	Failure (n = 18)	*p-value*
Age (months)	2.00 [1.00; 6.00]	3.00 [1.00; 6.00]	2.00 [1.25; 3.00]	.55^§^
**Sex**				
Male	46 (55.4)	39 (60.0)	7 (38.9)	.18^¶^
Female	37 (44.6)	26 (40.0)	11 (61.1)	
Weight at admission (kg)	5.70	5.80	4.95	.11^§^
	[4.50; 7.55]	[4.50; 7.80]	[4.50; 5.60]	
PIM 2 score—severity (0–100%)	0.16	0.16	0.16	.73^§^
	[0.16; 020]	[0.16; 020]	[0.16; 020]	
M-WCAS score	4.00	4.00	4.00	.50^§^
	[3.00; 5.00]	[3.00; 5.00]	[3.00; 5.00]	
Nasogastric tube during HFNC use	33 (39.8%)	19 (29.2%)	14 (77.8%)	< .001^¶^
**Equipment model**				.41^¶^
Optiflow (Fisher & Paykel)	53 (63.9%)	43 (66.2%)	10 (55.6%)	
Precision flow (Vapotherm)	30 (36.1%)	22 (33.8%)	8 (44.4%)	
Duration of HFNC use (hours)	46.00 [25.87; 70.50]	56.75 [40.00; 74.67]	12.75 [8.87; 23.69]	< .001^§^
Use of midazolam	5 (6.0)	0 (0.0)	5 (27.8)	< .001^†^
Use of fentanyl	4 (4.8)	0 (0.0)	4 (22.2)	< .002^†^

p-values for the chi-square test^(¶)^, Fisher’s exact test^(†)^ and Mann–Whitney test^(§)^.

*PIM 2* pediatric index of mortality, *M-WCAS* modified Wood's clinical asthma score, *HFNC* high-flow nasal cannula.

In the comparison of groups that used and did not use the nasogastric tube during HFNC use, we observed evidence of differences in terms of age (*p* = 0.031) and patients’ weight at admission (*p* = 0.003), in which the group that did not use the nasogastric tube had higher mean age and weight than the group with the use of the nasogastric tube (Table 2). The association of the occurrence of failure of the HFNC with the use of the nasogastric tube was investigated using a logistic model controlling for age. This adjustment was conducted to control the association observed between age and nasogastric tube use. Measures of age and weight were strongly correlated (r = 0.784; p < 0.001), and therefore, these two measures were not included in the same model. We chose age for inclusion in the model.

**Table 2:** General profile of patients and profile according to the use of nasogastric tube during HFNC.

	Total (n = 83)	Nasogastric tube during HFNC		p-value
		No (n = 50)	Yes (n = 33)	
Age (months)	2.00 [1.00; 6.00]	3.00 [1.00; 6.00]	2.00 [1.00; 3.00]	0.03
PIM 2 score—severity (0–100%)	0.16 [0.16; 0.20]	0.16 [0.16; 0.20]	0.16 [0.16; 0.19]	0.66
M-WCAS score	4.00 [3.00; 5.00]	4.00 [3.00; 5.00]	4.00 [3.00; 5.00]	0.88
Weight upon admission (kg)	5.70 [4.50; 7.60]	6.50 [4.60; 8.00]	5.00 [4.00; 5.70]	.003

p-values for Mann–Whitney test. Median values and interquartile range.

*PIM 2* pediatric index of mortality, *M-WCAS* modified Wood's clinical asthma score, *HFNC* high-flow nasal cannula.

Table 3 shows the results of univariate and multivariate logistic regression model approaches by evaluating the associations between failure and factors of interest. The results of logistic models showed that for patients requiring a nasogastric tube during HFNC use, the chances of failure were more likely than for patients with oral nutrition (OR = 8.17; 95% CI 2.30–28.99; *p* = 0.001).

**Table 3:** Results of approach univariate and multivariate logistic models assessing associations between failure and factors of interest.

Variables	Sucess failure		Univariate logistic model		Multivariate logistic model	
	(n = 65)	(n = 18)	OR (IC 95%)	*p-value*	OR (IC 95%)	*p-value*
**Nasogastric tube during HFNC use**				.001		.001
No	46 (70.8%)	4 (22.2%)	1,0		1,00	
Yes	19 (29.2%)	14 (77.8%)	8.47 (2.47; 29.08)		8.17 (2.30; 28.99)	
**Equipment model**				0.41		
Optiflow (Fisher & Paykel)	43 (66.2%)	10 (55.6%)	1.00			
Precision Flow (Vapotherm)	22 (33.8%)	8 (44.4%)	1.56 (0.54; 4.52)			
**Time between hospital admission and start of HFNC (hours)**				0.73		
Median (Q1; Q3)	6.5 (3.5; 18.8)	9.7 (3.9; 20.0)	1.01 (0.98; 1.03)			
**Age (month)**				0.29		0,818
Median (Q1; Q3)	3.0 (1.0; 6.0)	2.0 (1.0; 3.0)	0.91 (0.77; 1.08)		0,98 (0,82; 1,17)	

Age was included in the multivariate logistic model to control the observed association between age and use of a nasogastric tube (Table 2).

*OR* odds ratios, 95% confidence intervals for odds ratios (95% CI) and p-value, *HFNC* high-flow nasal cannula.

The HFNC failure was not associated with the device used, time between hospital admission and HFNC installation (OR = 1.01; 95% CI 0.98–1.03; *p* = 0.731), or age (OR = 0.91; 95% CI 0.77–1.08; *p* = 0.290). A total of 10 (18.8%) patients used the Optiflow system, and 8 (26.6%) individuals used the Precision Flow system (OR = 1.56; 95% CI 0.54–4.52; *p* = 0.41).

**Figure 1:**
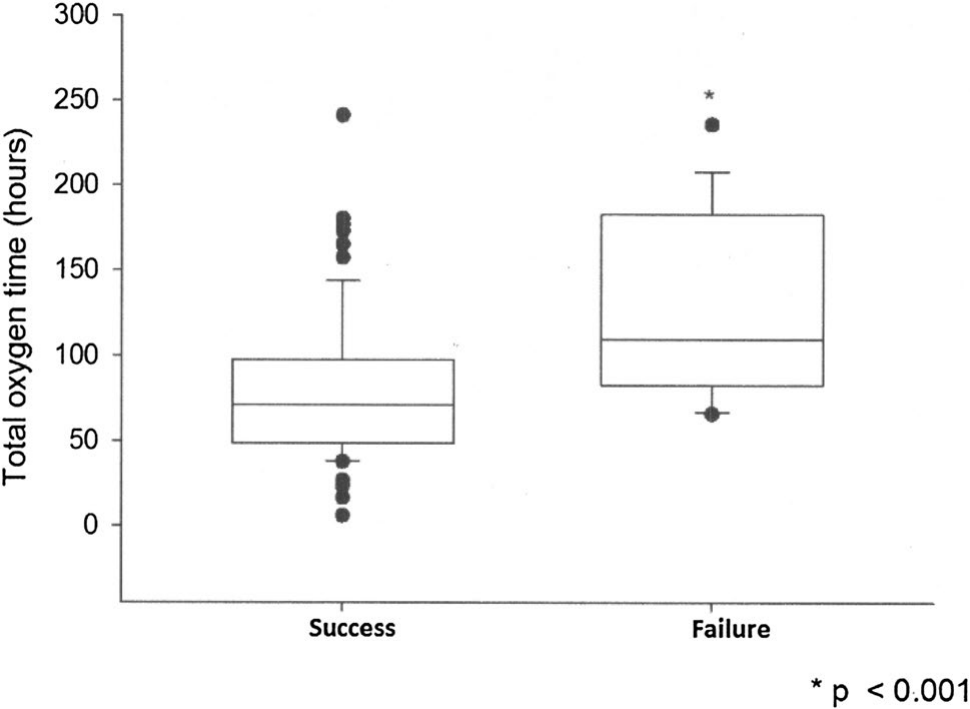
Difference in the total duration of oxygen use between the HFNC therapy success and failure groups. Values are presented as the median (horizontal line), 25–75% interquartile range (box) and upper and lower nonoutlier limits (vertical line). *p*-values are for the Mann–Whitney test.

**Figure 2:**
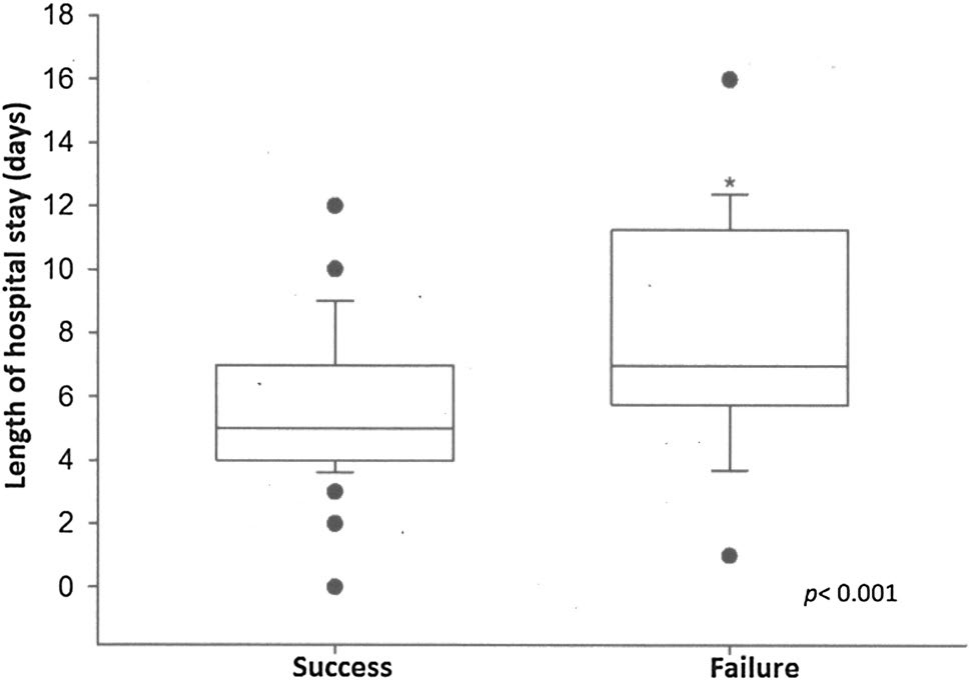
Difference in the total length of hospital stays between the HFNC therapy success and failure groups. Values are presented as the median (horizontal line), 25–75% interquartile range (boxplot) and upper and lower nonoutlier limits (vertical line). *p*-values are for the Mann–Whitney test.

Among the late outcomes evaluated, the patients with therapy failure had longer total durations of O_2_ use (*p* < 0.001) (Fig. 1) and longer hospital stays (*p* < 0.001) (Fig. 2).

## Discussion

Our study sought to determine risk factors associated with failure of HFNC therapy and found that the need to use the nasogastric tube during HFNC use was an important clinical marker for HFNC therapy failure.

Recently, some studies have initiated a discussion about whether to use oral nutrition in infants with bronchiolitis using HFNC, but none of them associated the need to use a nasogastric tube and HFNC therapy failure^17,21,22^.

In our study, the infants requiring a nasogastric tube during HFNC for feeding had higher risk of HFNC therapy failure. Our protocol of nasogastric tube use indication is based on clinical evaluation by the physician according to the severity of respiratory failure. The use of the nasogastric tube was indicated when the oral ingestion was sufficient and/or risk of coughing and bronchoaspiration were determined through the physician assessment.

Sochet et al. reported that oral nutrition in infants with bronchiolitis was well tolerated and that the nasogastric tube was required for infants with higher RR^17^, and other studies have shown the difficulty with feeding and changes in swallowing that can occur among infants with bronchiolitis, showing that interference of respiratory discomfort can affect the efficacy of suction and the swallowing process^23,24^. Therefore, the need for a nasogastric tube may be a marker of the severity of respiratory distress.

One possible confounding factor could be the effect of the patients' age and the need for a nasogastric tube. In our study, there was an association between patient age and weight at admission with the need for a nasogastric tube, but after the adjustment of multivariate analysis of the age of patients, there was no influence of this variable upon the use of the nasogastric tube as a predictor of HFNC failure; other authors found similar results to our study^13,15^. On the other hand, Suessman et al. observed a higher risk of intubation in breastfed babies younger than 2–3 months of age^18^.

There was no difference in the percentage of HFNC failure when two HFNC delivery systems were compared. Weiler et al. used the same two devices adopted in our study. The result was similar, and failure of therapy showed no association with the type of equipment used^19^.

In our study, the duration of HFNC use by the patients with HFNC therapy failure was lower than that in the patients for whom therapy was successful (12.8 h vs. 56.8 h). The same trend was described by Betters et al.^13^ (5.5 h vs. 28.0 h). Other authors mention that the durations of HFNC use among patients with therapy failure were 7 h and 14 h, which are similar to our results^15,16^. Studies related to NIV failure have shown that patients who did not have short-term results (1 to 2 h) were at increased risk of NIV failure^25,26^ and that the delay in interrupting NIV may have been associated with increased mortality^27,28^.

The importance of finding a marker for HFNC failure is that this can result in earlier detection, avoiding muscle fatigue and deterioration of the respiratory system.

Regarding the late outcomes, our study observed a shorter duration of oxygen use and a shorter hospital stay in the patients with successful HFNC therapy. Er et al.^14^ also found a difference in the lengths of hospital stays between patients who responded and did not respond to HFNC therapy. In contrast, with these findings, studies comparing HFNC therapy with conventional oxygen therapy found no difference in the lengths of hospital stays or the durations of oxygen use^15,29^, suggesting that the impact on these outcomes may be associated with the need for more advanced support, such as CPAP, bilevel positive airway pressure (BIPAP) or IMV.

Our study has the implicit limitations of a retrospective study. Another limitation of this study is the small number of patients with HFNC failure during the study period. Although 83 patients were included, only 18 had HFNC failure, which limited the power of the analyses. To date, studies evaluating risk factors for HFNC therapy failure have shown similar profiles to that found in our study. A total of 18 infants were observed in the sample of the failed group, 14 infants in Betters’ study^13^, and 8 in Mayfield’s study^30^. Overall, the percentage of HFNC failure among infants with bronchiolitis is low, and this result directly reflects sample size.

In conclusion, the need to use a nasogastric tube during HFNC use was associated with HFNC therapy failure, and this association can be considered as a marker of severity in children with bronchiolitis and serves as an alert parameter for early staging for other ventilatory support.
